# Supplementary material for: Heat adaptation of phage T7 under an extended genetic code
Source: Virus Evol. 2021 Dec 1;7(2):veab100. doi: 10.1093/ve/veab100 (PMC8923235; doi:10.1093/ve/veab100)
Supplement: veab100_Supp [file veab100_supp.zip › Sequencing_Analysis.zip!/Sequencing_Analysis/S2/evidence/RA_36.html]

BRESEQ :: Evidence


| Read alignment evidence... | | | | | | | | | | | |
| --- | --- | --- | --- | --- | --- | --- | --- | --- | --- | --- | --- |
|  | seq id | position | | ref | new | freq | score (cons/poly) | reads | annotation | genes | product |
| \* | NC\_001604.1 | 36,918 | 0 | T | C | 31.2% | 5428.1 / inf | 5123 | intergenic (–/–) | *–/–* | –/– |
| Reads supporting (aligned to +/- strand):  **ref** base T (1742/1760);  **new** base C (792/796);  **total** (2545/2578) | | | | | | | | | | | |
| Fisher's exact test for biased strand distribution *p*-value = 9.52e-01 | | | | | | | | | | | |
| Kolmogorov-Smirnov test that lower quality scores support variant *p*-value = 1.00e+00 | | | | | | | | | | | |

Only 100 of 5131 total aligned reads displayed.  

`GCTTGCGGACCTTTCGGACCATGAGCGTCGCTCTCCGCAACTCTATAATGCTATTAACAAACTGTTAGACCGCCACAAGTTCCAGATTGGTAAGTTGCAGCCGGATGTTCACATCTTAGGTGGCCTTGCTGGTGCTCTTGAAGAGTACAAAGAGAAAGTCGGTGATAACGGTCTTACGGATGATGATATTTACACATTACAGTGATATACTCAAGGCCACTACAGATAGTGGTCTTTATGGATGTCATTGTCTATACGAGATGCTCCTACGTGAAATCTGAAAGTTAACGGGAGGCATTATGCTAGAATTTTTACGTAAGCTAATCCCTTGGGTTCTCGCTGGGATGCTATTCGGGTTAGGATGGCATCTAGGGTCAGACTCAATGGACGCTAAATGGAAACAGGAGGTACACAATGAGTACGTTAAGAGAGTTGAGGCTGCGAAGAGCACTCAAAGAGCAATCGATGCGGTATCTGCTAAGTATCAAGAAGACCTTGCCGCGCTGGAAGGGAGCACTGATAGGATTATTTCTGATTTGCGTAGCGACAATAAGCGGTTGCGCGTCAGAGTCAAAACTACCGGAACCTCCGATGGTCAGTG  >  NC_001604.1/36618‑37218`  

                                                                                                                                                                                                                                                                                                            `|`                                                                                                                                                                                                                                                                                                              
   `tGCGGACCTTTCGGACCATGAGCGTCGCTCTCCGCAACTCTATAATGCTATTAACAAACTGTTAGACCGCCACAAGTTCCAGATTGGTAAGTTGCAGCTGGATGTTCACATCTTAGGTGGCCTTGCTGGTGCTCTTGAAGAGTACAAAGAGAAAGTCGGTGATAACGGTCTTACGGATGATGATATTTACACATTACAGTGATATACTCAAGGCAACTACAGATAGTGGTCTTTATGGATGTCATTGTCTATACGAGATGCTCCTACGTGAAATCTGAAAGTTAACGGGAGGCATTATGCt                                                                                                                                                                                                                                                                                                           <  1:621351/301‑1 (MQ=255)`  
           `tttCGGACCATGAGCGTCGCTCTCCGCAACTCTATAATGCTATTAACAAACTGTTAGACCGCCACAAGTTCCAGATTGGTAAGTTGCAGCCGGATGTTCACATCTTAGGTGGCCTTGCTGGTGCTATTGAAGAGTACAAAGAGAAAGTCGGTGATAACGGTCTTACGGATGATGATATTTACACATTACAGTGATATACTCAAGGCCACTACAGATAGTGGTCTTTATGGATGTCATTGTCTATACGAGATGCTCCTACGTGAAATCTGAAAGTTAACGGGAGGCATTACGCTAGAAtttt                                                                                                                                                                                                                                                                                                   >  1:772574/1‑301 (MQ=255)`  
                           `tCGCTCTCCGCAACTCTATAATGCTATTAACAAACTGTTAGACCGCCACAAGTTCCAGATTGGTAAGTTGCAGCCGGATGTTCACATCTTAGGTGGCCTTGCTGGTGCTCTTGAAGAGTACAAAGAGAAAGTCGGTGATAACGGTCTTACGGATGATGATATTTACACATTACAGTGATATACTCAAGGCCACTACAGATAGTGGTCTTTATGGATGTCATTGTCTATACGAGATGCTCCTACGTGAAATCTGAAAGTTAACGGGATGCATTATGCTAGAATTTTTACGTAAGCTAATccc                                                                                                                                                                                                                                                                                   <  1:115026/301‑1 (MQ=255)`  
                                  `ccGCAACTCTATAATGCTATTAACAAACTGTTAGACCGCCACAAGTTCCAGATTGGTAAGTTGCAGCCGGATGTTCACATCTTAGGTGGCCTTGCTGGTGCTCTTGAAGAGTACAAAGAGAAAGTCGGTGATAACGGTCTTACGGATGATGATATTTACACATTACAGTGATATACTCAAGGCCACTACAGATAGTGGTCTTTATGGATGTCATTGTCTATACGAGATGCTCCTACGTGAAATCTGAAAGTTAACGGGAGGCATTACGCTAGAATTTTTACGTAAGCTAATCCCtgtctct                                                                                                                                                                                                                                                                            >  1:213844/1‑295 (MQ=255)`  
                                       `aCTCTATAATGCTATTAACAAACTGTTAGACCGCCACAAGTTCCAGATTGGTAAGTTGCAGCCGGATGTTCACATCTTAGGTGTCCTTGCTGGTGCTCTTGAAGAGTACAAAGAGAAAGTCGGTGATAACGGTCTTACGGATGATGATATTTACACATTACAGTGATATACTCAAGGCCACTACAGATAGTGGTCTTTATGGATGTCATTGTCTATACGAGATGCTCCTACGTGAAATCTGAAAGTTAACGGGAGGCATTACGCTAGAATTTTTACGTAAGCTAATCCCTTGGGTTCTCGc                                                                                                                                                                                                                                                                       >  1:322006/1‑301 (MQ=255)`  
                                       `aCTCTATAATGCTATTAACAAACTGTTAGACCGCCACAAGTTCCAGATTGGTAAGTTGCAGCCGGATGTTCACATCTTAGGTGGCCTTGCTGGTGCTCTTGAAGAGTACAAAGAGAAAGTCGGTGATAACGGTCTTACGGATGATGATATTTACACATTACAGTGATATACTCAAGGCCACTACAGATAGTGGTCTTTATGGATGTCATTGTCTATACGAGATGCTCCTACGTGAAATCTGAAAGTTAACGGGAGGCATTACGCTAGAATTTTTACGTAAGCTAATCCCTTGGGTTCTCGc                                                                                                                                                                                                                                                                       >  1:509536/1‑301 (MQ=255)`  
                                       `aCTCTATAATGCTATTAACAAACTGTTAGACCGCCACAAGTTCCAGATTGGTAAGTTGCAGCCGGATGTTCACATCTTAGGTGGCCTTGCTGGTGCTCTTGAAGAGTACAAAGAGAAAGTCGGTGATAACGGTCTTACGGATGATGATATTTACACATTACAGTGATATACTCAAGGCCACTACAGATAGTGGTCTTTATGGATGTCATTGTCTATACGAGATGCTCCTACGTGAAATCTGAAAGTTAACGGGAGGCATTACGCTAGAATTTTTACGTAAGCTAATCCCTTGGGTTCTCGc                                                                                                                                                                                                                                                                       >  1:729086/1‑301 (MQ=255)`  
                                          `ctATAATGCTATTCACAAACTGTTAGACCGCCACAAGTTCCAGATTGGTAAGTTGCAGCCGGATGTTCACATCTTAGGTGGCCTTGCTGGTGCTCTTGAAGAGTACAAAGAGAAAGTCGGTGATAACGGTCTTACGGATGATGATATTTACACATTACAGTGATATACTCAAGGCCACTACAGATAGTGGTCTTTATGGATGTCATTGTCTATACGAGATGCTCCTACGTGAAATCTGAAAGTTAACGGGAGGCATTACGCTAGAATTTTTACGTAAGCTAATCCCTTTGGTTCCCGCTgg                                                                                                                                                                                                                                                                    >  1:765494/1‑301 (MQ=255)`  
                                          `ctATAATGCTATTAACAAACTGTTAGACCGCCACAAGTTCCAGATTGGTAAGTTGCAGCCGGATGTTCACATCTTAGGTGGCCTTGCTGGTGCTCTTGAAGAGTACAAAGAGAAAGTCGGTGATAACGGTCTTACGGATGATGATATTTACACATTACAGTGATATACTCAAGGCCACTACAGATAGTGGTCTTTATGGATGTCATTTTCTATACGAGATGCTCCTCGGTGAAATCTGAAAGTTAACGGGGGGAATTACGCTAGAATTTTTACGTAAGCTAACCCCTTGGGTTctggctgg                                                                                                                                                                                                                                                                    >  1:744406/1‑301 (MQ=255)`  
                                          `ctATAATGCTATTAACAAACTGTTAGACCGCCACAAGTTCCAGATTGGTAAGTTGCAGCCGGATGTTCACATCTTAGGTGGCCTTGCTGGTGCTCTTGAAGAGTACAAAGAGAAAGTCGGTGATAACGGTCTTACGGATGATGATATTTACACATTACAGTGATATACTCAAGGCCACTACAGATAGTGGTCTTTATGGATGTCATTTTCTATACGAGATGCTCCTACGTGAAATCTGAAAGTTAACGGGAGGCATTACGCTAGAATTTTTACGTAAGCTAATCCCTTgggctgtctctta                                                                                                                                                                                                                                                                    >  1:20756/1‑291 (MQ=255)`  
                                               `aTGCTATTAACAAACTGTTAGACCGCCACGAGTTCCAGATTGGTAAGTTGCAGCCGGATGTTCACATCTTAGGTGGCCTTGCTGGTGCTCTTGAAGAGTACAAAGAGAAAGTCGGTGATAACGGTCTTACGGATGATGATATTTACACATTACAGTGATATACTCAAGGCCACTACAGATAGTGGTCTTTATGGATGTCATTGTCTATACGAGATGCTCCTACGTGAAATCTGAAAGTTAACGGGAGGCATTATGCTAGAATTTTTACGTAAGCTAATCCCTTGGGTTCTCGCTGGGATGc                                                                                                                                                                                                                                                               <  1:221001/301‑1 (MQ=255)`  
                                                  `cTATTAACAAACTGTTAGACCGCCACAAGTTCCAGATTGGTAAGTTGCAGCCGGATGTTCACATCTTAGGTGGCCTTGCTGGTGCTCTTGAAGAGTACAAAGAGAAAGTCGGTGATAACGGTCTTACGGATGATGATATTTACACATTACAGTGATATACTCAAGGCCACTACAGATAGTGGTCTTTATGGATGTCATTGTCTATACGAGATGCTCCTACGTGAAATCTGAAAGTTAACGGGAGGCATTACGCTAGAATTTTTACGTAAGCTAATCCCTTGGGTTCTCGCTGGGATGCTAt                                                                                                                                                                                                                                                            >  1:298242/1‑301 (MQ=255)`  
                                                       `aaCAAACTGTTAGACCGCCACAAGTTCCAGATTGGTAAGTTGCAGCCGGATGTTCACATCTTAGGTGGCCTTGCTGGTGCTCTTGAAGAGTACAAAGAGAAAGTCGGTGATAACGGTCTTACGGATGATGATATTTACACATTACAGTGATATACTCAAGGCCACTACAGATAGTGGTCTTTATGGATGTCATTGTCTATACGAGATGCTCCTACGTGAAATCTGAAAGTTAACGGGAGGCATTACGCTAGAATTTTTACGTAAGCTAATCCCTTGGGTTCTCGCTGGGATGCTATTCggg                                                                                                                                                                                                                                                       <  1:563828/301‑1 (MQ=255)`  
                                                         `cAAACTGTTAGACCGCCACAAGTTCCAGATTGGTAAGTTGCAGCCGGATGTTCACATCTTAGGTGGCCTTGCTGGTGCTCTTGAAGAGTACAAAGAGAAAGTCGGTGATAACGGTCTTACGGATGATGATATTTACACATTACAGTGATATACTCAAGGCCACTACAGATAGTGGTCTTTATGGATGTCATTGTCTATACGAGATGCTCCTACGTGAAATCTGAAAGTTAACGGGAGGCATTATGCTAGAATTTTTACGTAAGCTAATCCCTTGGGTTCTCGCTGGGATGCTATTCGGGtt                                                                                                                                                                                                                                                     <  1:596954/301‑1 (MQ=255)`  
                                                         `aaaaCAGTAAGACCGCCACAAGTTCAAGATTGGTAAGTTGCAGCCGGATGTTCACAACTTAGGTGGCCTTGCTGGTGCTATTGAAGAGTACAAAGAGAAAGACGGTGATAACGGTCTTACGGATGATGATAATTACACATTAAAGTGATATACTAAAGGCCACTACAGATAGTGGTCTTTAAGGATGTCATTGTCTATACGAGATGCTCATAAGTGAAATCTGAAAGTGAACGGGAGGCATTATGCTAGAATTTTTACGTAAGCTAATCCCTTGGGTTCTCGCTGGGATGCTATTCGGGtt                                                                                                                                                                                                                                                     <  1:173812/300‑1 (MQ=255)`  
                                                             `cTGTTAGACCGCCACAAGTTCCAGATTGGTAAGTTGCAGCCGGATGTTCACATCTTAGGTGGCCTTGCTGGTGCTCTTGAAGAGTACAAAGAGAAAGTCGGTGATAAAGGTCTTACGGATGATGATATTTACACATTACAGTGATATACTCAAGGCCACTACAGATAGTGGTCTTTATGGATGTCATTGTCTATACGAGATGCTCCTACGTGAAATCTGAAAGTTAACGGGAGGCATTACGCTAGAATTTTTACGTAAGCTAATCCCTTGGGTTCTCGCTGGGATGCTATTCGGGTTAGGa                                                                                                                                                                                                                                                 <  1:805001/301‑1 (MQ=255)`  
                                                              `cggagatgtgtataagagacaggATTGGTAAGTTGCAGCCGGATGTTCACATCTTAGGTGGCCTTGCTGGTGCTCTTGAAGAGTACAAAGAGAAAGTCGGTGATAACGGTCTTACGGATGATGATATTTACACATTACAGTGATATACTCAAGGCCACTACAGATAGTGGTCTTTATGGATGTCATTGTCTATACGAGATGCTCCTACGTGAAATCTGAAAGTTAACGGGAGGCATTACGCTAGAATTTTTACGTAAGCTAATCCCTTGGGTTTTCGCTGGGATGCTATTCGGGTTAGGAt                                                                                                                                                                                                                                                <  1:57711/279‑1 (MQ=255)`  
                                                                   `gACCGCCACAAGTTCCAGATTGGTAAGTTGCAGCCGGATGTTCACATCTTAGGTGGCCTTGCTGGTGCTCTTGAAGAGTACAAAGAGAAAGTCGGTGATAACGGTCTTACGGATGATGATATTTACACATTACAGTGATATACTCAAGGCCACTACAGATAGTGGTCTTTATGGATGTCATTGTCTATACGAGATGCTCCTACGTGAAATCTGAAAGTTAACGGGAGGCATTATGCTAGAATTTTTACGTAAGCTAATCCCTTGGGTTCTCGCTGGGATGCTATTCGGGTTAGGATGGCAt                                                                                                                                                                                                                                           <  1:159285/301‑1 (MQ=255)`  
                                                                       `gCCACAAGTTCCAGATTGGTAAGTTGCAGCCGGATGTTCACATCTTAGGTGGCCTTGCTGGTGCTCTTGAAGAGTACAAAGAGAAAGTCGGTGATAACGGTCTTACGGATGATGATATTTAAACATTACAGTGATATACTCAAGGCCACTACAGATGGTGGTCTTTATGGATGTCATTGTCTATACGAGATGCTCCTACGTGAAATCGGAAAGTTAACGGGAGGAATTATGCTAGAATTTTTGCGTAAGCTAATCCCTTGGGTTCTCGCTGGGATGCTATTCGGGTTAGGATGGCATCTAg                                                                                                                                                                                                                                       >  1:243720/1‑301 (MQ=255)`  
                                                                              `gTTCCAGATTGGTAAGTTGCAGCTGGATGTTCACATCTTAGGTGGCCTTGCTGGTGCTCTTGAAGAGTACAAAGAGAAAGTCGGTGATAACGGTCTTACGGATGATGATATTTACACATTACAGTGATATACTCAAGGCCACTACAGATAGTGGTCTTTATGGATGTCATTGTCTATACGAGATGCTCCTACGTGAAATCTGAAAGTTAACGGGAGGCATTATGCTAGAATTTTTACGTAAGCTAATCCCTTGGGTTCTCGCTGGGATGCTATTCGGGTTAGGATGGCATCTAGGGTCAGa                                                                                                                                                                                                                                >  1:426552/1‑301 (MQ=255)`  
                                                                                        `ggTAAGTTGCAGCCGGATGTTCACATCTTAGGTGGCCTTGCTGGTGCTCTTGAAGAGTACAAAGAGAAAGTCGGTGATAACGGTCTTACGGATGATGATATTTACACATTACAGTGATATACTCAAGGCCACTACAGATAGTGGTCTTTATGGATGTCATTGTCTATACGAGATGCTCCTACGTGAAATCTGAAAGTTAACGGGAGGCATTATGCTAGAATTTTTACGTAAGCTAATCCCTTGGGTTCTCGCTGGGATGCTATTCGGGTTAGGATGGCATCTAGGGTCAGACTCAATGGAc                                                                                                                                                                                                                      <  1:100432/301‑1 (MQ=255)`  
                                                                                        `ggTAAGTTGCAGCCGGATGTTCACATCTTAGGTGGCCTTGCTGGTGCTCTTGAAGAGTACAAAGAGAAAATCGGTGATAACGGTCTTACGGATGATGATATTTACACATTACAGTGATATACTCAAGGCCACTACAGATAGTGGTCTTTATGGATGTCATTGTCTATACGAGATGCTCCTACGTGAAATCTGAAAGTTAACGGGAGGCATTATGCTAGAATTTTTACGTAAGCTAATCCCTTGGGTTCTCGCTGGGATGCTATTCGGGTTAGGATGGCATCTAGGGTCAGACTCAATGGAc                                                                                                                                                                                                                      >  1:195516/1‑301 (MQ=255)`  
                                                                                             `gTTGCAGCCGGATGTTCACATCTTAGGTGGCCTTGCTGGTGCTCTTGAAGAGTACAAAGAGAAAGTCGGTGATAACGGTCTTACGGATGATGATATTTACACATTACAGTGATATACTCAAGGCCACTACAGATAGTGGTCTTTATGGATGTCATTGTCTATACGAGATGCTCCTACGTGAAATCTGAAAGTTAACGGGAGGCATTATGCTAGAATTTTTACGTAAGCTAATCCCTTGGGTTCTCGCTGGGATGCTATTCTGGTTAGGATGGCATCTAGGGTCAGACTCAATGGACGCTaa                                                                                                                                                                                                                 >  1:706111/1‑301 (MQ=255)`  
                                                                                                       `gATGTTCACATCTTAGGTGGCCTTGCTGGTGCTCTTGAAGAGTACAAAGAGAAAGTCGGTGATAACGGTCTTACGGATGATGATATTTACACATTACAGTGATATACTCAAGGCCACTACAGATAGTGGTCTTTATGGATGTCATTGTCTATACGAGATGCTCCTACGTGAAATCTGAAAGTTAACGGGAGGCATTACGCTAGAATTTTTACGTAAGCTAATCCCTTGGGTTCTCGCTGGGATGCTATTCGGGTTAGGATGGCATCTAGGGTCAGACTCAATGGACGCTAAATGGAAACag                                                                                                                                                                                                       >  1:441264/1‑301 (MQ=255)`  
                                                                                                        `aTGTTCACATCTTAGGTGGCCTTGCTGGTGCTCTTGAAGAGTACAAAGAGAAAGTCGGTGATAACGGTCTTACGGATGATGATATTTACACATTACAGTGATTTACTCAAGGCCACTACAGATAGTGGTCTTTATGGATGTCATTGTCTATACGAGATGCTCCTACGTGAAATCTGAAAGTTAACGGGAGGCATTATGCTAGAATTTTTACGTAAGCTAATCCCTTGGGTTCTCGCTGGGATGCTATTCGGGTTAGGATGGCATCTAGGGTCAGACTCAATGGACGCTAAATGGAAACagg                                                                                                                                                                                                      <  1:711509/301‑1 (MQ=255)`  
                                                                                                                              `tGCTGGTGCTCTTGAAGAGTACAAAGAGAAAGTCGGTGATAACGGTCTTACGGATGATGATATTTACACATTACAGTGATATACTCAAGGCCACTACAGATAGTGGTCTTTATGGATGTCATTGTCTATACGAGATGCTCCTACGTGAAATCTGAAAGTTAACGGGAGGCATTATGCTAGAATTTTTACGTAAGCTAATCCCTTGGGTTCTCGCTGGGATGCTATTCGGGTTAGGATGGCATCTAGGGTCAGACTCAATGGACGCTAAATGGAAACAGGAGGTACACAATGAGTACGTTaa                                                                                                                                                                                >  1:463210/1‑301 (MQ=255)`  
                                                                                                                                 `tGGTGCTCTTTAAGATTACAAAGAGAAAGTCGGTGATAACGGTCTTACGGATGATGATATTTACACATTACAGTGATATACTCAAGGCCACTACAGATAGTGGTCTTTATGGATGTCATTGTCTATACGAGATGCTCCTACGTGAAATCTGAAAGTTAACGGGAGGCATTACGCTAGAATTTTTACGTAAGCTAATCCCTTGGGTTCTCGCTGGGATGCTATTCGGGTTAGGATGGCATCTAGGGTCAGACTCAATGGACGCTAAATGGAAACAGGAGGTACACAATGAGTACGTTAagag                                                                                                                                                                             <  1:86193/301‑1 (MQ=255)`  
                                                                                                                                   `gTGCTCTTGAAGAGTACAAAGAGAAAGTCGGTGATAACGGTCTTACGGATGATGATATTTACACATTACAGTGATATACTCAAGGACACTACAGATAGTGGTCTTTATGGATGTCATTGTCTATACGAGATGCTCCTCCGTGAAATCTGAAAGTTAACGGGAGGCATTATGCTAGAATTTTTACGTAAGCTAATCCCTTGGGTTCTCGCTGGGATGCTATTCGGGTTAGGATGGCATCTAGGGTCAGACTCAATGGACGCAAAATGGAAACAGGAGGTACACAATGAGTACGTTAagagag                                                                                                                                                                           >  1:340201/1‑301 (MQ=255)`  
                                                                                                                                      `ctctTGAAGAGTACAAAGAGAAAGTCGGTGATAACGGTCTTACGGATGATGATATTTACACATTACAGTGATATACTCAAGGCCACTACAGATAGTGGTCTTTATGGATGTCATTGTCTATACGAGATGCTCCTACGTGAAATCTGAAAGTTAACGGGAGGCATTACGCTAGAATTTTTACGTAAGCTAATCCCTTGGGTTCTCGCTGGGATGCTATTCGGGTTAGGATGGCATCTAGGGTCAGACTCAATGGACGCTAAATGGAAACAGGAGGTACACAATGAGTACGTTAAGAGAGTTg                                                                                                                                                                        <  1:448022/301‑1 (MQ=255)`  
                                                                                                                                                `gTACAAAGAGAAAGTCGGTGATAACGGTCTTACGGATGATGATATTTACACATTACAGTGATATATTCAAGGCCACTACAGATAGTGGTCTTTATGGATGTCATTGTCTATACGAGATGCTACTACGTGAAATCTGAAAGTTAACGGGAGGCATTACGCTAGAATTTTTACGTAAGCTAATCCCTTGGGTTATCGCTGGGATGCTATTCGGGTTAGGATGGCATTTAGGGTCAGACTCAATGGACGCTAAATGGAAACAGGAGGTACACAATGAGTACGTTAAGAGAGTTGAGGCTGCGaa                                                                                                                                                              >  1:421119/1‑301 (MQ=255)`  
                                                                                                                                                `gTACAAAGAGAAAGTCGGTGATAACGGTCTTACGGATGATGATATTTACACATTACAGTGATATACTCAAGGCCACTACAGATAGTGGTCTTTATGGATGTCATTGTCTATACGAGATGCTCCTACGTGAAATCTGAAAGTTAACGGGAGGCATTACGCTAGAATTTTTACGTAAGCTAATCCCTTGGGTTCTCGCTGGGATGCTATTCGGGTTAGGATGGCATCTAGGGTCAGACTCAATGGACGCTAAATGGAAACAGGAGGTACACAATGAGTACGTTAAGAGAGTTGAGGCTGCGaa                                                                                                                                                              >  1:257055/1‑301 (MQ=255)`  
                                                                                                                                                `gTACAAAGAGAAAGTCGGTGAAAACGGTCTTACGGATGATGATATTTACACATTACAGTGATATACTCAAGGCCACTACAGATAGTGGTCTTTATGGATGTCATTGTCTATACGAGATGCTCCTACGTGAAATCTGAAAGTTAACGGGAGGCATTATGCTAGAATTTTTACGTAAGCTAATCCCTTGGGTTCTCGCTGGGATGCTATTCGGGTTAGGATGGCATCTAGGGTCAGACTCAATGGACGCTAAATGGAAACAGGAGGTACACAATGAGTACGTTAAGAGAGTTGAGGCTGCGaa                                                                                                                                                              >  1:306932/1‑301 (MQ=255)`  
                                                                                                                                                      `agagAAAGTCGGTGATAACGGTCTTACGGATGATGATATTTACACATTACAGTGATATACTCAAGGCCACTACAGATAGTGGTCTTTATGGATGTCATTGTCTATACGAGATGCTCCTACGTGAAATCTGAAAGTTAACGGGAGGCATTATGCTAGAATTTTTACGTAAGCTAATCCCTTGGGTTCTCGCTGGGATGCTATTCGGGTTAGGATGGCATCTAGGGTCAGACTCAATGGACGCTAAATGGAAACAGGAGGTACACAATGAGTACGTTAAGAGAGTTGAGGCTGCGAAGAGCAc                                                                                                                                                        <  1:674444/301‑1 (MQ=255)`  
                                                                                                                                                                        `cGGTCTTACGGATGATGATATTTACACATTACAGTGATATACTCAAGGCCACTACAGATAGTGGTCTTTATGGATGTCATTGTCTATACGAGATGCTCCTACGTGAAATCTGAAAGTTAACGGGAGGCATTACGCTAGAATTTTTACGTAAGCTAATCCCTTGGGTTCTCGCTGGGATGCTATTCGGGTTAGGATGGCATCTAGGGTCAGACTCAATGGACGCTAAATGGAAACAGGAGGTACACAATGAGTACGTTAAGAGAGTTGAGGCTGCGAAGAGCACTCAAAGAGCAATCGATGc                                                                                                                                      >  1:667165/1‑301 (MQ=255)`  
                                                                                                                                                                         `ggTCTTACTGATGATGATATTTACACATTACAGTGATATACTCAAGGCCACTACAGATAGTGGTCTTTATTGTTGTCATTGTCTTTACGAGATGCTCCTACGTGAAATCTGAAAGTTCACGGGAGGCATTATGCTAGAATTTTTACGTAAGCTAATCCCTTGGGTTCTCGCTGGGATGCTATTCGGGTTAGGATGGCATCTAGGGTCAGACTCACTGGACGCTAAATGGAAACAGGAGGTACACAATGAGTACGTTAAGAGAGTTGAGGCTGCGAAGAGCACTCAAAGAGCAATCGATGCg                                                                                                                                     <  1:590998/301‑1 (MQ=255)`  
                                                                                                                                                                          `gTCTTACGGATGATGATATTTACACATTACAGTGATATACTCAAGGCCACTACAGATAGTGGTCTTTATGGATGTCATTGTCTATACGAGATGCTCCTACGTGAAATCTGAAAGTTAACGGGAGGCATTACGCTAGAATTTTTACGTAAGCTAATCCCTTGGGTTCTCGCTGGGATGCTATTCGGGTTAGGATGGCATCTAGGGTCAGACTCAATGGACGCTAAATGGAAACAGGAGGTACACAATGAGTACGTTAAGAGAGTTGAGGCTGCGAAGAGCACTCAAAGAGCAATCGATGCgg                                                                                                                                    >  1:201837/1‑301 (MQ=255)`  
                                                                                                                                                                             `ttACGGATGATGATATTTACACATTACAGTTATATACTCAAGGCCACTACAGATAGTGGTCTTTATGGAAGTCATTGTCTATACGAGATGCTCCTACGTGAAATCTGAAAGTTAACGGGAGGCATTACGCTAGAATTTTTACGTAAGCTAATCCCTTGGGTTCTCGCTGGGATGCTATTCGGGTTAGGATGGCATCTAGGGTCAGACTCAATGGACGCTAAATGGAAACAGGAGGTACACAATGAGTACGTTAAGAGAGTTGAGGCTGCGAAGAGCACTCAAAGAGCAATCGATGCGGTAt                                                                                                                                 <  1:570866/301‑1 (MQ=255)`  
                                                                                                                                                                             `ttACGGATGATGATATTTACACATTACAGTGATATACTCAAGGCCACTACAGATAGTGGTCTTTATGGATGTCATTGTCTATACGAGATACTCCTACGTGAAATCTGAAAGTTAACGGGAGGCATTATAGTAGGATTTTTACGTAGGCTAATGCCTTGGGTTCTCGCTGGGATGCTATTCGGGTTAGGATGGCATCTAGGGTCAGACTCAATGGACGCTAAATGGAAACAGGAGGTACACAATGAGTACGTTAAGAGAGTTGAGGCTGCGAAGAGCACTCAAAGAGCAATCGATGCGGTAt                                                                                                                                 <  1:41089/301‑1 (MQ=255)`  
                                                                                                                                                                             `gagacagtgatgatATTTACACATTACAGTGATATACTCAAGGCCACTACAGATAGTGGTCTTTATGGATGTCATTGTCTATACGAGATGCTCCTACGTGAAATCTGAAAGTTAACGGGAGGCATTACGCTAGAATTTTTACGTAAGCTAATCCCTTGGGTTCTCGCTGGGATGCTATTCGGGTTAGGATGGCATCTAGGGTCAGACTCAATGGACGCTAAATGGAAACAGGAGGTACACAATGAGTACGTTAAGAGAGTTGAGGCTGCGAAGAGCACTCAAAGAGCAATCGATGCGGTAt                                                                                                                                 <  1:823779/294‑1 (MQ=255)`  
                                                                                                                                                                                  `tatgatgatATTTACACATTACAGTGATATACTCAAGGCCACTACAGATAGTGGTCTTTATGGATGTCATTGTCTATACGAGATGCTCCTACGTGAAATCTGAAAGTTAACGGGAGGCATTATGCTAGAATTTTTACGTAAGCTAATCCCTTGGGTTCTCGCTGGGATGCTATTCGGGTTAGGATGGCATCTAGGGTCAGACTCAATGGACGCTAAATGGAAACAGGAGGTACACAATGAGTACGTTAAGAGAGTTGAGGCTGCGAAGAGCACTCAAAGAGCAATCGATGCGGTATCTGCt                                                                                                                            <  1:796835/300‑1 (MQ=255)`  
                                                                                                                                                                                  `gatgatgatATTTACACATTACAGTGATATACTCAAGGCCACTACAGATAGTGGTCTTTATGGATGTCATTGTCTATACGAGATGCTCCTACGTGAAATCTGAAAGTTAACGGGAGGCATTATGCTAGAATTTTTACATAAGCTAATCCCTTGGGTTCTCGCTGGGATGTTATTCGGGTTAGGATGGCATCTAGTGTCAGACTCAATGGACGCTAAATGGAAACAGGAGGTACACAATGAGTACGTTAAGAGAGTTGAGGCTGCGAAGAGCACTCAAAGAGCAATCGCTgtctcttataca                                                                                                                            >  1:347998/1‑290 (MQ=255)`  
                                                                                                                                                                                  `gatgatgatATTTACACATTACAGTGATATACTCAAGGCCACTACAGATAGTGGTCTTTATGGATGTCATTGTCTATACGAGATGCTCCTACGTGAAATCTGAAAGTTAACGGGAGGCATTACGCTAGAATTTTTACGTAAGCTAATCCCTTGGGTTCTCGCTGGGATGCTATTCGGGTTAGGATGGCATCTAGGGTCAGACTCAATGGACGCTAAATGGAAACAGGAGGTACACAATGAGTACGTTAAGAGAGTTGAGGCTGCGAAGAGCACTCAAAGAGCAATCGATGCGGTactgtct                                                                                                                            >  1:251177/1‑295 (MQ=255)`  
                                                                                                                                                                                   `atgatgatATTTACACATTACAGTGATATACTCAAGGCCACTACAGATAGTGGTCTTTATGGATGTCATTGTCTATACGAGATGCTCCTACGTGAAATCTGAAAGTTAACGGGAGGCATTACGCTAGAATTTTTACGTAAGCTAATCCCTTGGGTTCTCGCTGGGATGCTATTCGGGTTAGGATGGCATCTAGGGTCAGACTCAATGGACGCTAAATGGAAACAGGAGGTACACAATGAGTACGTTAAGAGAGTTGAGGCTGCGAAGAGCACTCAAAGAGCAATCGATGCGGTATCTGCTa                                                                                                                           <  1:269436/301‑1 (MQ=255)`  
                                                                                                                                                                                     `gatgatATTTACACATTACAGTGATATACTCAAGGCCACTACAGATAGTGGTCTTTATGGATGTCATTGTCTATACGAGATGCTCCTACGTGAAATCTGAAAGTTAACGGGAGGCATTATGCTAGAATTTTTACGTAAGCTAATCCCTTGGGTTCTCGCTGGGATGCTATTCGGGTTAGGATGGCATCTAGGGTCAGACTCAATGGACGCTAAATGGAAACAGGAGGTACACAATGAGTACGTTAAGAGAGTTGAGGCTGCGAAGAGCACTCAAAGAGCAATCGATGCGGTATCTGCTAAg                                                                                                                         <  1:690650/301‑1 (MQ=255)`  
                                                                                                                                                                                      `agatgtgtataagagataggtgagatcatCAAGGCCGCTATAGATAGTGGGCTTTATGGATGTAATTGTCTATACGAGATGCTACTACGTGAATTCTGAAAGTTAACGGGGGGCATTATGCTAGAAATTTTACGTAAGCTAAGCCCTTGGGTTCTCCCTGGGATGCTATTCGGGTTAGGATGGCATCTAGGGTCAGACTCAATGGACGCTAAATGGAAACAGGAGGTACACAATGAGTACGTTAAGAGAGTTGAGGCTGCGAAGTGCACTCAAAGAGCAATCGATGCGGTATCTGCTAAGt                                                                                                                        <  1:354607/273‑1 (MQ=255)`  
                                                                                                                                                                                      `aataagagaaaacaTAACAATGATATACTCAAGGCCACTACAGATAGTGGGCTTTATGGATGTCATTGTCTATACGAGATGCTCCTACTTGAAATCTGAAAGTTAACGGGAGGCATTACGATAGAATTTTTACGTAAGCTAATCCCTTGGGTGCTCGCTGGGATGCTAATCGGGTTAGGATGGCATCTAGGGTCAGACTCAATGGACGCTAAAGGGAAACAGGAGGTACACAATGAGTACGTTAAGAGAGTTGAGGCTGCGAAGAGCACTCAAAGAGCAATCGATGCGGTATCTGCTAAGt                                                                                                                        <  1:107542/290‑1 (MQ=255)`  
                                                                                                                                                                                        `agatgtttataaaagacaggttataCTCAAGGCCACTACAGATAGTGGTCTTTATGGATGTCATTGTCTATCCGCGATGCTCCTACGTGAAATCTGAAAGTTAACGGGAGGCATTACGCTAGAATTTTTACGTAAGCTAATCCCTTGGGTTCTCGCTGGGATGCTATTCGGGTTAGGATGGCATCTAGGGTCAGACTCAATGGACGCTAAATGGAAACAGGAGGTACACAATGAGTACGTTAAGAGAGTTGAGGCTGCGAAGAGCACTCAAAGAGCAATCGATGCGGTATCTGCTAAGTAt                                                                                                                      <  1:780613/280‑1 (MQ=255)`  
                                                                                                                                                                                          `tatTTACACATTACAGTGATATACTCAAGGCCACTACAGATAGTGGTCTTTATGGATGTCATTGTCTATACTAGATGCTCCTACGTGAAATCTGGAAGTTAACGGGAGGCATTCTGCTAGAATTTTTACTTCAGCTACTCCCTTGGGTTCTCGCTGGGATGCTATTCGGGTTAGGATGGCATCTAGGGTCAGACTCAATGGACGCTAAATGGAAACAGGAGGTACACAATGAGTACGTTAAGAGAGTTGAGGCTGCGAAGAGCACTCAAAGCGCAATCGATGCGGTATCTGCTAAGTATCa                                                                                                                    >  1:330606/1‑301 (MQ=255)`  
                                                                                                                                                                                          `tatTTACACATTACAGTGATATACTCAAGGCCACTACAGATAGTGGTCTTTATGGATGTCATTGTCTATACGAGATGCTCCTACGTGAAATCTGAAAGTTAACGGGAGGCATTATGCTAGAATTTTTACGTAAGCTAATCCCTTGGGTTCTCGCTGGGATGCTATTCGGGTTAGGATGGCATCTAGGGTCAGACTCAATGGACGCTAAATGGAAACAGGAGGTACACAATGAGTACGTTAAGAGAGTTGAGGCTGCGAAGAGCACTCAAAGAGCAATCGATGCGGTATCTGCTAAGTATCa                                                                                                                    >  1:49504/1‑301 (MQ=255)`  
                                                                                                                                                                                          `tatTTACACATTACAGTGATATACTCAAGGCCACTACAGATAGTGGTCTTTATGGATGTCATTGTCTATACGAGATGCTCCTACGTGAAACCTGAAAGTTAACGGGAGGCATTATGCTAGAATTTTTACGTAAGCTAATCCCTTGGGTTCTCGCTGGGATGCTATTCGGGTTAGGATGGCATCTAGGGTCAGACTCAATGGACGCTAAATGGAAACAGGAGGTACACAATGAGTACGTTAAGAGAGTTGAGGCTGCGAAGAGCACTCAAATAGCAATCGATGCGGTATCTGCTAAGTATCa                                                                                                                    >  1:315454/1‑301 (MQ=255)`  
                                                                                                                                                                                             `ttACACATTTCAGTGATATACTCAAGGCCACTACAGATAGTTGTCTTTATGGATGTCATTGTCTATACGAGATTCTCCTACGTGAAATCTGAAAGTTAACGGGAGGCATTATGCTAGAATTTTTACGTAAGCTAAGCCCTTGGGTTCTCGCTGGGATGCTATTCGGGTTAGGATGGCATCTAGGGTCAGACTCAATGGACGCTAAATGGAAACAGGAGGTACACAATGAGTACGTTAAGAGAGTTGAGGCTGCGAAGAGCACTCAAAGAGCAATCGATGCGGTATCTGCTAAGTATCaaga                                                                                                                 <  1:540990/301‑1 (MQ=255)`  
                                                                                                                                                                                             `ttACACATTACAGTGATATACTCAAGGCCACTACAGATAGTGGTCTTTATGGATGTCATTGTCTATACGAGATGCTCCTACGTGAAATCTGAAAGTTAACGGGAGGCATTATGCTAGAATTTTTACGTAAGCTAATCCCTTGGGTTCTCGCTGGGATGCTATTCGGGTTAGGATGGCATCTAGGGTCAGACTCAATGGACGCTAAATGGAAACAGGAGGTACACAATGAGTACGTTAAGAGAGTTGAGGATGCGAAGAGCACTCAAAGAGCAATCGATACGGTATCTGCTAAGTATCaaga                                                                                                                 >  1:93120/1‑301 (MQ=255)`  
                                                                                                                                                                                             `gctcggagaagtgtctaagggacagagCCACTACAGATAGTGGTCTTTATGGAGGTCAGTGTCTAGACGAGATGCGCCTACGTGAAATCTGAAAGTTAACGGGAGGCATTATGCTAGAATTTTTACGTAAGCTAATCCCTTGGGTGCTCGCTGGGATGCTATTCGGGTTAGGATGGCATCTAGGGTCAGACTCAATGGACGCTAAATGGAAACAGGAGGTACACAATGAGTACGTTAAGAGAGTTGAGGCTGCGAAGAGCACTCAAAGAGCAATCGATGCGGTATCTGCTAAGTATCaaga                                                                                                                 <  1:236452/275‑1 (MQ=255)`  
                                                                                                                                                                                             `gagatgtgtataagagacagcTCAAGGCCACTACAGATAGTGGTCTTTATGGATGTCATTGTCTATACGAGATGCTCCTACGTGAAATCTGAAAGTTAACGGGAGGCATTACGCTAGAATTTTTACGTAAGCTAATCCCTTGGGTTCTCGCTGGGATGCTATTCGGGTTAGGATGGCATCTAGGGTCAGACTCAATGGACGCTAAATGGAAACAGGAGGTACACAATGAGTACGTTAAGAGAGTTGAGGCTGCGAAGAGCACTCAAAGAGCAATCGATGCGGTATCTGCTAAGTATCaaga                                                                                                                 <  1:187878/281‑1 (MQ=255)`  
                                                                                                                                                                                             `aagagacagaCAGTGATATACTCAAGGCCACTACAGATAGTGGTCTTTATGGATGTCATTGTCTATACGAGATGCTCCTACGTGAAATCTGAAAGTTAACGGGAGGCATTATGCTAGAATTTTTACGTAAGCTAATCCCTTGGGTTCTCGCTGGGATGCTATTCGGGTTAGGATGGCATCTAGGGTCAGACTCAATGGACGCTAAATGGAAACAGGAGGTACACAATGAGTACGTTAAGAGAGTTGAGGCTGCGAAGAGCACTCAAAGAGCAATAGATGCGGTATCTGCTAAGTATCaaga                                                                                                                 <  1:143353/292‑1 (MQ=255)`  
                                                                                                                                                                                                  `caTTACAGTGATATACTCAAGGCCACTACAGATAGTGGTCGTTATGTATGTCATTGTCTATACGAGATGCTCCTACGTGAAATCTGAAAGTTAACGGGAGGCATTATGCTAGAATTTTTACGTAAGCTAATCCCTTGGGTTTTCGCTGGGATGCCATTCGGGTTAGGATGGCACCTAGGGTCAGACTCAATGGACGCTAAATGGAAACAGGAGGTACACAATGAGTACGTTAAGAGAGTTGAGGCTGCGAAGAGCACTCAAAGAGCAATCGATGCGGTATCTGCTAAGTATCAAGACGAcc                                                                                                            >  1:485514/1‑301 (MQ=255)`  
                                                                                                                                                                                                       `cAGTGATATACTCAAGGCCACTACAGATAGTGGTCTTTATGGATGTCATTGTCTATACGAGATGCTCCTACGTGAAATCTGAAAGTTAACGGGAGGCATTATGCTAGAATTTTTACGTAAGCTAATCCCTTGGGTTCTCGCTGGGATGCTATTCGGGTTAGGATGGCATCTAGGGTCAGACTCAATGGACGCTAAATGGAAACAGGAGGTACACAATGAGTACGTTAAGAGAGTTGAGGCTGCGAAGAGCACTCAAAGAGCAATCGATGCGGTATCTGCTAAGTATCAAGAAGACCTTGcc                                                                                                       >  1:386928/1‑301 (MQ=255)`  
                                                                                                                                                                                                           `gATATACTCAAGGCCACTACAGATAGTGGTCTTTATGGATGTCATTGTCTATACGAGATGCTCCTACGTGAAATCTGAAAGTTAACGGGAGGCATTATGCTAGAATTTTTACGTAAGCTAATCCCTTGGGTTCTCGCTGGGATGCTATTCGGGTTAGGATGGCATCTAGGGTCAGACTCAATGGACGCTAAATGGAAACAGGAGGTACACAATGAGTACGTTAAGAGAGTTGAGGCTGCGAAGAGCACTCAAAGAGCAATCGATGCGGTATCTGCTAAGTATCAAGAAGACCTTGCCgcgc                                                                                                   >  1:395090/1‑301 (MQ=255)`  
                                                                                                                                                                                                           `gATATACTCAAGGCCACTACAGATAGTGGTCTTTATGGATGTCATTGTCTATACGAGATGCTCCTACGTGAAATCTGAAAGTTAACGGGAGGCATTACGCTAGAATTTTTACGTAAGCTAATCCCTTGGGTTCTCGCTGGGATGCTATTCGGGTTAGGATGGCATCTAGGGTCAGACTCAATGGACGCTAAATGGAAACAGGAGGTACACAATGAGTACGTTAAGAGAGTTGAGGCTGCGAAGAGCACTCAAAGAGCAATCGATGCGGTATCTGCTAAGTATCAAGAAGACCTTGCCgcgc                                                                                                   >  1:180912/1‑301 (MQ=255)`  
                                                                                                                                                                                                              `ataCTCAAGGCCACTACAGATAGTGGTCTTTATGGATGTCATTGTCTATACGAGATGCTCCTACGTGAAATCTGAAAGTTAACGGGAGGCATTATGCTAGAATTTTTACGTAAGCTAATCCCTTGGGTTCTCGCTGGGATGCTATTCGGGTTAGGATGGCATCTAGGGTCAGACTCAATGGACGCTAAATGGAAACAGGAGGTACACAATGAGTACGTTAAGAGAGTTGAGGCTGCGAAGAGCACTCAAAGAGCAATCGATGCGGTATCTGCTAAGTATCAAGAAGACCTTGCCGAGCTgg                                                                                                >  1:810291/1‑301 (MQ=255)`  
                                                                                                                                                                                                                `aCTCAAGGCCACTACAGATAGTGGTCTTTATGGATGTCATTGTCTATACGAGATGCTCCTACGTGAAATCTGAAAGTTAACGGGAGGCATTATGCTAGAATTTTTACGTAAGCTAATCCCTTGGGTTCTCGCTGGGATGCTATTCGGGTTAGGATGGCATCTAGGGTCAGACTCAATGGACGCTAAATGGAAACAGGAGGTACACAATGAGTACGTTAAGAGAGTTGAGGCTGCGAAGAGCACTCAAAGAGCAATCGATGCGGTATCTGCTAAGTATCAAGAAGACCTTGCCGCGCTGGaa                                                                                              >  1:377469/1‑301 (MQ=255)`  
                                                                                                                                                                                                                 `cTCAAGGCCACTACAGATAGTGGTCTTTATGGATGTCATTGTCTATACGAGATGCTCCTACGTGAAATCTGAAAGTTAACGGGAGACATTATGCTAGAATTTTTACGTAAGCTAATCCCTTGGGTTCTCGCTGGGATGCTATTCGGGTTAGGATGGCATCTAGGGTCAGACTCAATGGACGCTAAATGGAAACAGGAGTTACACAATGAGTACGTTAAGAGAGTTGAGGCTGCGAAGAGCACTCAAAGAGCAATCGATGCGGTATCTGCTAAGTATCAAGAAGACCTTGCCGCGCTGGAAg                                                                                             <  1:584468/301‑1 (MQ=255)`  
                                                                                                                                                                                                                 `cTCAAGGCCACTACAGATAGTGGTCTTTATGGATGTCATTGTCTATACCAGATGCTCCTACGTGAAATCTGAAAGTTAACGGGAGGCATTATGCTAGAATTTTTACGTAAGCTAATCCCTTGGGTTCTCGCTGGGATGCTATTCGGGTTAGGATGGCATCTAGGGTCAGACTCAATGGACGCTAAATGGAAACAGGAGGTACACAATGAGTACGTTAAGAGAGTTGAGGCTGCGAAGAGCACTCAAAGAGCAATCGATGCGGTATCTGCTAAGTATCAAGAAGACCTTGCCGCGCTGGAAg                                                                                             <  1:736866/301‑1 (MQ=255)`  
                                                                                                                                                                                                                   `cagggcccatACAGATAGTGGTCTTTATGGATGTCATTGTCTATACGAGATGCTCCTACGTGAAATCTGAAAGTTAACGGGAGGCATTACGCTAGAATTTTTACGTAAGCTAATCCCTTGGGTTCTCGCTGGGATGCTATTCGGGTTAGGATGGCATCTAGGGTCAGACTCAATGGACGCTAAATGGAAACAGGAGGTACACAATGAGTACGTTAAGAGAGTTGAGGCTGCGAAGAGCACTCAAAGAGCAATCGATGCGGTATCTGCTAAGTATCAAGAAGACCTTGCCGCGCTGGAAggg                                                                                           <  1:628723/292‑1 (MQ=255)`  
                                                                                                                                                                                                                       `gCCACTACAGATAGTGGTCTTTATGGATGTCATTGTCTATACGAGATGCTCCTACGTGAAATCTGAAAGTTAACGGGAGGCATTATGCTAGAATTTTTACGTAAGCTAATCCCTTGGGTTCTCGCTGGGATGCTATTCGGGTTAGGATGGCATCTAGGGTCAGACTCAATGGACGCTAAATGGAAACAGGAGGTACACAATGAGTACGTTAAGAGAGTTGAGGCTGCGAAGAGCACTCAAAGAGCAATCGATGCGGTATCTGCTAAGTATCAAGAAGACCTTGCCGCGCTGGAAGGGAGCa                                                                                       >  1:651481/1‑301 (MQ=255)`  
                                                                                                                                                                                                                           `cTACAGATAGTGGTCTTTATGGATGTCATTGTCTATACGAGATGCTCCTACGTGAAATCTGAAAGTTAACGGGAGGCATTATGCTAGAATTTTTACGTAAGCTAATCCCTTGGGTTCTCGCTGGGATGCTATTCGGGTTAGGATGGCATCTAGGGTCAGACTCAATGGACGCTAAATGGAAACAGGAGGTACACAATGAGTACGTTAAGAGAGTTGAGGCTGCGAAGAGCACTCAAAGAGCAATCGATGCGGTATCTGCTAAGTATCAAGAAGACCTTGCCGCGCTGGAAgctgtctctta                                                                                   >  1:532436/1‑291 (MQ=255)`  
                                                                                                                                                                                                                           `cTACAGATAGTGGTCTTTATGGATGTCATTGTCTATACGAGATGCTCCTACGTGAAATCTGAAAGTTAACGGGAGGCATTATGCTAGAATTTTTACGTAAGCTAATCCCTTGGGTTCTCGCTGGGATGCTATTCGGGTTAGGATGGCATCTAGGGTCAGACTCAATGGACGCTAAATGGAAACAGGAGGTACACAATGAGTACGTTAAGAGAGTTGAGGCTGCGAAGAGCACTCAAAGAGCAATCGATGCGGTATCTGCTAAGTATCAAGAAGACCTTGCCGCGCTGGAAGGGAGCACTGa                                                                                   >  1:477351/1‑301 (MQ=255)`  
                                                                                                                                                                                                                           `cTACAGATAGTGGTCTTTATGGATGTCATTGTCTATACGAGATGCTCCTACGTGAAATCTGAAAGTTAACGGGAGGCATTACGCTAGAATTTTTACGTAAGCTAATCCCTTGGGTTCTCGCTGGGATGCTATTCGGGTTAGGATGGCATCTAGGGTCAGACTCAATGGACGCTAAATGGAAACAGGAGGTACACAAAGAGTACGTTAAGAGAGTTGAGGCTGCGAAGAGCACTCAAAGAGCAATCGATGCGGTATCTGCTAAGTATCAAGAAGACCTTGCCGCGCTGGAAGGGAGCACTGa                                                                                   >  1:453994/1‑301 (MQ=255)`  
                                                                                                                                                                                                                              `cAGATAGTGGTCTTTATGGATGTCATTGTCTATACGAGATGCTCCTACATGAAATCTGAAAGTTAACGGGAGGCATTATGCTAGAATTTTTACGTAAGCTAATCCCTTAGGTTCTCGCTGGGATGCTATTCGGGTTAGGATGGCATCTAGGGTCAGACTCAATGGACGCTAAATGGAAACAGTAGGTACACAATGAGTACGTTAAGAGAGTTGAGGCTGCGAAGAGCACTCAAAGAGCAATCGATGCGGTATCTGCTAAGTATCAAGAAGACCTTGCCGCGCTGGAAGGGAGCACTGATAg                                                                                >  1:289344/1‑301 (MQ=255)`  
                                                                                                                                                                                                                                `gATAGTGGTCTTTATGGATGTCATTGTCTATACGAGATGCTCCTACGTGAAATCTGAAAGTTAACGGGAGGCATTATGCTAGAATTTTTACGTAAGCTAATCCCTTGGGTTCTCGCTGGGATGCTATTCGGGTTAGGATGGCATCTAGGGTCAGACTCAATGGACGCTAAATGGAAACATGAGGTACACAATGAGTACGTTAAGAGAGTTGAGGCTGCGAAGAGCACTCAAAGAGCAATCGATGCGGTATCTGCTAAGTATCAAGAAGACCTTGCCGCGCTGGAAGGGAGCACTGATAGGa                                                                              <  1:229428/301‑1 (MQ=255)`  
                                                                                                                                                                                                                                `gATAGTGGTCTTTATGGATGTCATTGTCTATACGAGATGCTCCTACGTGAAATCTGAAAGTTAACGGGAGGCATTATGCTAGAATTTTTACGTAAGCTAATCCCTTGGGTTCTCGCTGGGATGCTATTCGGGTTAGGATGGCATCTAGGGTCAGACTCAATGGACGCTAAATGGAAACAGGAGGTACACAATGAGTACGTTAAGAGAGTTGAGGCTGCGAAGAGCACTCAAAGAGCAATCGATGCGGTATCTGCTAAGTATCAAGAAGACCTTGCCGCGCTGGAAGGGAGCACTGATAGGa                                                                              <  1:121117/301‑1 (MQ=255)`  
                                                                                                                                                                                                                                `aagagacagcTTTATGGATGTCATGGTCTATACGAGATGCTCATACGTGAAATCTGAAAGTTAACGGGAGGCATTACGCTAGAATTTTTACGTAAGCTAATCCCTTGGGTTCTCGATGGGATGCTATTCGGGTTAGGATGGCATCTAGGGTCAGACTCAATGGAAGCTAAATGGAAACAGGAGGTACACAATGAGTACGTTAAGAGAGTTGAGGCTGCGAAGAGCACTCAAAGAGCAATCGATGCGGTATCTGCTAAGTATCAAGAAGACCTTGCCGCGCTGGAAGGGAGCACTGATAGGa                                                                              <  1:263384/292‑1 (MQ=255)`  
                                                                                                                                                                                                                                `aaTAGTGGTCTTTATGGATGTCATTGTCTATACGAGATGCTCCTACGTGAAATCTGAAAGTTAACGGGAGGCATTATGCTAGAATTTTTACGTAAGCTAATCCCTTGGGTTCTCGCTGGGATGCTATTCGGGTTAGGATGGCATCTAGGGTCAGACTCAATGGACGCTAAATGGAAACAGGAGGTACACAATGAGTACGTTAAGAGAGTTGAGGCTGCGAAGAGCACTCAAAGAGCAATCGATGCGGTATCTGCTAAGTATCAAGAAGACCTTGCCGCGCTGGAAGGGAGCACTGATAGGa                                                                              <  1:40160/300‑1 (MQ=255)`  
                                                                                                                                                                                                                                 `aTAGTGGTCTTTATGGATGTCATTGTCTATACGAGATGCTCCTACGTGAAATCTGAAAGTTAACGGGAGGCATTATGCTAGAATTTTTACGTAAGCTAATCCCTTGGGTTCTCGCTGGGATGCTATTCGGGTTAGGATGGCATCTAGGGTCAGACTCAATGGACGCTAAATGGAAACAGGAGGTACACAATGAGTACGTTAGGAGAGTTGAGGCTGCGAAGAGCACTCAAAGAGCAATCGATGCGGTATCTGCTAAGTATCAAGAAGACCTTGCCGCGCTGGACGGGAGCACTGATAGGat                                                                             >  1:127732/1‑301 (MQ=255)`  
                                                                                                                                                                                                                                 `aTAGTGGTCTTTATGGATGTCATTGTCTATACGAGATGCTCCTACGTGAAATCTGAAAGTTAACGGGAGGCATTATGCTAGAATTTTTACGTAAGCTAATCCCTTGGGTTCTCGCTGGGATGCTATTCGGGTTAGGATGGCATCTAGGGTCAGACTCAATGGACGCTAAATGGAAACAGGAGGTACACAATGAGTACGTTAAGAGAGTTGAGGCTGCGAAGAGCACTCAAAGAGCAATCGATGCGGTATCTGCTAAGTATCAAGAAGACCTTGCCGCGCTGGAAGGGAGCACTGATAGGac                                                                             >  1:433589/1‑300 (MQ=255)`  
                                                                                                                                                                                                                                       `gtatgtATGGATGTCATTGTCTATACGAGATGCTCCTACGTGAAATCTGAAAGTTAACGGGAGGCATTACGCTAGAATTTTTACGTAAGCTAATCCCTTGGGTTCTCGCTGGGATGCTATTCGGGTTAGGATGGCATCTAGGATCAGACTCAATGGACGCTAAATGGAAACAGGAAGTACACAATGAGTACGTTAAGAGAGTTGAGGCTGCGAAGAGCACTCAAAGAGCAATCGATGCGGTATCTGCTAAGTATCAAGAAGACCTTGCCGCGCTGGAAGGGAGCACTGATAGGATTATTTc                                                                       <  1:698299/296‑1 (MQ=255)`  
                                                                                                                                                                                                                                       `gTCTTTATGGATGTCATTGTCTATACGAGATGCTCCTACGTGAAATCTGAAAGTTAACGGGAGGCATTATGCTAGAATTTTTACGTAAGCTAATCCCTTGGGTTCTCGCTGGGATGCTATTCGGGTTAGGATGGCATCTAGGGTCAGACTCAATGGACGCTAAATGGAAACAGGAGGTACACAATGAGTACGTTAAGAGAGTTGAGGCTGCGAAGAGCACTCAAAGAGCAATCGATGCGGTATCTGCTAAGTATCAAGAAGACCTTGCCGCGCTGGAAgggctgtctcttatacacatctc                                                                       >  1:362672/1‑281 (MQ=255)`  
                                                                                                                                                                                                                                       `gTCTTTATGGATGTCATTGTCTATACGAGATGCTCCTACGTGAAATCTGAAAGTTAACGGGAGGCATTACGCTAGAATTTTTACGTAAGCTAATCCCTTGGGTTCTCGCTGGGATGCTATTCGGGTTAGGATGGCATCTAGGGTCAGACTCAATGGACGCTAAATGGAAACAGGAGGTACACAATGAGTACGTTAAGAGAGTTGAGGCTGCGAAGAGCACTCAAAGAGCAATCGATGCGGTATCTGCTAAGTATCAAGAAGACCTTGCCGCGCTGGAAGGGAGCACTGATAGGATTATTTc                                                                       <  1:515048/301‑1 (MQ=255)`  
                                                                                                                                                                                                                                       `gTCTTTAGTGATGTCATTTTCTATACGAGATGCTCCTACGTGAAATCTGAAAGTTAACGGGAGGCATTATGCTAGAATTTTTACGTAAGCTAATCCCTTGGGTTCTCGCTGGGATGCTATTCGGGTTAGGATGGCATCTAGGGTCAGACTCAATGGACGCTAAATGGAAACAGGAGGTACACAATGAGTACGTTAAGAGAGTTGAGGCTGCGAAGAGCACTCAAAGAGCAATCGATGCGGTATCTGCTAAGTATCAAGAAGACCTTGCCGCGCTGGAAGGGAGCACTGATAGGATTATTTc                                                                       <  1:63634/301‑1 (MQ=255)`  
                                                                                                                                                                                                                                           `ttATGGATGTCATTGTCTATACGAGATGCTCCTACGTGAAATCTGAAAGTTAACGGGAGGCATTACGCTAGAATTTTTACGTAAGCTAATCCCTTGGGTTCTCGCTGGGATGCTATTCGGGTTAGGATGGCATCTAGGGTCAGACTCAATGGACGCTAAATGGAAACAGGAGGTACACAATGAGTACGTTAAGAGAGTTGAGGCTGCGAAGAGCACTCAAAGAGCAATCGATGCGGTATCTGCTAAGTATCAAGAAGACCTTGCCGCGCTGGAAGGGAGCACTGATactgtctcttatacc                                                                   >  1:523616/1‑287 (MQ=255)`  
                                                                                                                                                                                                                                           `ttATGGATGTCATTGTCTATACGAGATGCTCCTACGTGAAATATGAAAGTTAACGGGAGGCATTATGCTAGAATTTTTACGTAAGCTAATCCCTTGGGTTCTCGCTGGGATGCTAGTCGGGTTAGGATGGCATCTAGGGTCAGACTCAATGGACGCTAAATGGAAACAGGAGGTACACAATGAGTACGTTAAGAGAGTTGAGGCTGCGAAGAGCACTCAAAGAGCAATCGATGCGGTATCTGCTAAGTATCAAGAAGACCTTGCCGCGCTGGAAGGGAGCACTGATAGGATTATTTCTGAt                                                                   <  1:658578/301‑1 (MQ=255)`  
                                                                                                                                                                                                                                                   `gTCATTGTCTATACGAGATGCTCCTACGTGAAATCTGAAAGTTAACGGGAGGCATTATGCTAGAATTTTTACGTAAGCTAATCCCTTGGGTTCTCGCTGGGATGCTATTCGGGTTAGGATGGCATCTAGGGTCAGACTCAATGGACGCTAAATGGAAACAGGAGGTACACAATGAGTACGTTAAGAGAGTTGAGGCTGCGAAGAGCACTCAAAGAGCAATCGATGCGGTATCTGCTAAGTATCAAGAAGACCTTGCCGCGCTGGAAGGGAGCACTGATAGGATTATTTCTGATTTGCGTAg                                                           >  1:165738/1‑301 (MQ=255)`  
                                                                                                                                                                                                                                                      `tataatagacagagaTGCTCATACGTGAAATCTGAAAGTTAACGGGAGGCATTATGCTAGAATTTTTACATAAGCTAATCCCTTGGGTTCTCGCTGGGATGCTATTCGGGTTAGGATGGCATCTAGGGTCAGACTCAATGGACGCTAAATGGAAACAGGAGGTACACAATGAGTACGTTAAGAGAGTTGAGGCTGCAAAGAGCACTCAAAGAGCAATCGATGCGGTATCTGCTAAGTATCAAGAAGACCTTGCCGCGCTGGAAGTGAGCACTGATAGGATTATTTCTGATTTGCGTAGCGa                                                        <  1:470464/290‑1 (MQ=255)`  
                                                                                                                                                                                                                                                      `aTTGTCTATACGAGATGCTCCTACGTGAAATCTGAAAGTTAACGGGAGGCATTATGCTAGAATTTTTACGTAAGCTAATCCCTTGGGTTCTCGCTGGGATGCTATTCGGGTTAGGATGGCATCTAGGGTCAGACTCAATGGACGCTAAATGGAAACAGGAGGTACACAATGAGTACGTTAAGAGAGTTGAGGCTGCGAAGAGCACTCAAAGAGCAATCGATGCGGTATCTGCTAAGTATCAAGAAGACCTTGCCGCGCTGGAAGGGAGCACTGATAGGATTATTTCTGATTTGCGTAGCGa                                                        <  1:15092/301‑1 (MQ=255)`  
                                                                                                                                                                                                                                                       `gatgttgataagagacagccTAGGTGAAATCTGAAAGTTAACGGGAGGCATTACGCTAGAATTTTTACGTAAGCTAATCCCTTGGGTTCTCGCTGGGATGCTATTCGGGTTAGGATGGCATCTAGGGTCAGACTCAATGGACGCTAAATGGAAACAGGAGGTACACAATGAGTACGTTAAGAGAGTTGAGGCTGCGAAGAGCACTCAAAGAGCAATCGATGCGGTATCTGCTAAGTATCAAGAAGACCTTGCCGCGCTGGAAGGGAGCACTGATAGGATTATTTCTGATTTGCGTAGCGAc                                                       <  1:789632/283‑1 (MQ=255)`  
                                                                                                                                                                                                                                                         `gTCTATACGAGATGCTCCTACGTGAAATCTGAAAGTTAACGGGAGGCATTATGCTAGAATTTTTACGTAAGCTAATCCCTTGGGTTCTCGCTGGGATGCTATTCGGGTTAGGATGGCATCTAGGGTCAGACTCAATGGACGCTAAATGGAAACAGGAGGTACACAATGAGTACGTTAAGAGAGTTGAGGCTGCGAAGAGCACTCAAAGAGCAATCGATGCGGTATCTGCTAAGTATCAAGAAGACCTTGCCGCGCTGGAAGGGAGCACTGATAGGATTATTTCTGATTTGCGTAGCGACaa                                                     >  1:644584/1‑301 (MQ=255)`  
                                                                                                                                                                                                                                                         `gTCTATACGAGATGCTCCTACGTGAAATCTGAAAGTTAACGGGAGGCATTATGCTAGAATTTTTACGTAAGCTAATCCCTTGGGTTCTCGCTGGGATGCTATTCGGGTTAGGATGGCATCTAGGGTCAGACTCAATGGACGCTAAATGGAAACAGGAGGTACACAATGAGTACGTTAAGAGAGTTGAGGATGCGAAGAGCACTCAAAGAGCAATCGATGCGGTATCTGCTAAGTATCAAGAAGACCTTGCCGCGCTGGAAGGGAGCACTGATAGGATTATTTCTGATTTGCGTAGCGACaa                                                     >  1:605781/1‑301 (MQ=255)`  
                                                                                                                                                                                                                                                           `atataCGAGATGCTCCTACGTGAAATCTGAAAGTTAACGGGAGGCATTACGCTAGAATTTTTACGTAAGCTAATCCCTTGGGTTCTCGCTGGGATGCTATTCGGGTTAGGATGGCATCTAAGGTCAGACTCAATGGACGCTAAATGGAAACAGGAGGTACACAATGAGTACGTTAAGAGAGTTGAGGCTGCGAAGAGCACTCAAAGAGCAATCGATGCGGTATCTGCTAAGTATCAAGAAGACCTTGCCGCGCTGGAAGGGAGCACTGATAGGATTATTTCTGATTTGCGTAGCGACAATa                                                   <  1:136524/300‑1 (MQ=255)`  
                                                                                                                                                                                                                                                            `tataCGAGATGCTCCTACGTGAAATCTGAAAGTTAACGGGAGGCATTACGCTAGAATTTTTACGTAAGCTAATCCCTTGGGTTCTCGCTGTGATGCTATTCGGGTTAGGATGGCATCT‑GGGTCAGACTCAATGGACGCTAAATGGAAACAGGAGGTACACAATGAGTACGTTAAGAGAGTTGAGGCTGCGAAGAGCACTCAAAGAGCAATCGATGCGGTATCTGCTAAGTATCAAGAAGACCTTGCCGCGCTGGAAGGGAGCACTGATAGGATTATTTCTGATTTGGGTAGCGACAATAAg                                                 <  1:719887/301‑1 (MQ=255)`  
                                                                                                                                                                                                                                                             `ataCGAGATGCTCCTACGTGAAATCTGAAAGTTAACGGGAGGCATTACGCTAGAATTTTTACGTAAGCTAATCCCTTGGGTTCTCGCTGGGATGCTATTCGGGTTAGGATGGCATCTAGGGTCAGACTCAATGGACGCTAAATGGAAACAGGAGGTACACAATGAGTACGTTAAGAGAGTTGAGGCTGCGAAGAGCACTCAAAGAGCAATCGATGCGGTATCTGCTAAGTATAAAGAAGACCTTGCCGCGCTGGAAGGGAGCACTGATAGGATTATTTCTGATTTGCGTAGCGACAATAAg                                                 >  1:750144/1‑301 (MQ=255)`  
                                                                                                                                                                                                                                                                 `gagaTGCTGTTACGTGAAATCTGAAAGTGAACGGGAGGCAGTACGCTAGAAGTTTTAGGTAAGTCAATCCCTTGGGTTCTCGCGGGGATGATATTCGGGTTAGGAGGGAATCTAGGGTCTGACTCAAGGGACGCTAAATGGAAACAGGAGGTACACAATGAGTACGTTAAGAGAGTTGAGGCTGCGAAGAGCACTCAAAGAGCAATCGATGCGGTATCTGCTAAGTATCAAGAAGACCTTGCCGCGCTGGAAGGGAGCACTGATAGGATTATTTCTGATTTGCGTAGCGACAATAAGCGGt                                             <  1:501573/301‑1 (MQ=255)`  
                                                                                                                                                                                                                                                                         `ccTACGTGAAATCTGAAAGTTAACGTGAGGCATTACGCTAGAATTTTTACGTAAGCTAATCCCTTGGGTTCTCGCTGGGATGCTATTCGGGTTAGGATGGCATCTAGGGTCAGACTCAATGGATGCTAAATGGAAACAGGAGGTACACAATGAGTACGTTAAGAGAGTTGAGGCTGCGAAGAGCACTCAAAGAGCAATCGATGCGGTATCTGCTAAGTATCAAGAAGACCTTGCCGCGCTGGAAGGGAGCACTGATAGGATTATTTCTGATTTGCGTAGCGACAATAAGCGGtctgtctct                                     >  1:554282/1‑293 (MQ=255)`  
                                                                                                                                                                                                                                                                           `tACGTGAAATCTGAAAGTTAACGGGAGGCATTACGCTAGAATTTTTACGTAAGCTAATCCCTTGGGTTCACGCTGGGATGCTATTCGGGTTAGGATGGCATCTAGGGTCAGACTCAATGGACGCTAAATGGAAACAGTAGGTACACAATGAGTACGTTAAGAGAGTTGAGGCTGCGAAGAGCACTCAAAGAGCAATCGATGCGGTATCTGCTAAGTATCAAGAAGACCTTGCCGCGCTGGAAGGGAGCACTGATAGGATTATTTCTGATTTGCGTAGCGACAATAAGCGGTTGCGCGTCag                                   <  1:37012/301‑1 (MQ=255)`  
                                                                                                                                                                                                                                                                             `cGTGAAAGCTGAAAGTTAACGGGAGGCATTATGCTAGAATTTTTACGTAAGCTAATCCCTTGGGTTCTCGCTGGGATGCTATTCGAGTTAGGATGGCATCTAGGGTCAGACTCAATGGACGCTAAATGGAAACAGGAGGTACACAATGAGTACGTTAAGAGAGTTGAGGCTGCGAAGAGCACTCAAAGAGCAATCGATGCGGTATCTGCTAAGTATCAAGAAGACCTTGCCGCGCTGGAAGGGAGAACTGATAGGATTATTTCTGATTTGCGTAGCGACAATAAGCGGTGGCGCGTCagag                                 <  1:276366/301‑1 (MQ=255)`  
                                                                                                                                                                                                                                                                                   `aTCTGAAAGTTAACGGGAGGCATTACGCTAGAATTTTTACGTAAGCTAATCCCTTGGGTTCTCGCTGGGATGCTATTCGGGTTAGGATGGCATCTAGGGTCAGACTCAATGGATGCTAAATGGAAACAGGAGGTACACAATGAGTACGTTAAGAGAGTTGAGGCTGCGAATAGCACTCAAAGAGCAATCGATGCGGTATCTGCTAAGTATCAAGAAGACCTTGCCGCGCTGGAAGGGAGCACTGATAGGATTATTTCTGATTTGCGTAGCGACAATAAGCGGTTGCGCGTCAGAGTCaaaa                           >  1:681813/1‑301 (MQ=255)`  
                                                                                                                                                                                                                                                                                          `aGTTAACGGGAGGCATTACGCTAGAATTTTTACGTAAGCTAATCCCTTGGGTTCTCGCTGGGATGCTATTCGGGTTAGGATGGCAGCTAGGGTCAGACTCAATGGACGCTAAATGGAAACAGGAGGTACACAATGAGTACGGTAAGAGAGTTGAGGATGCGAAGAGCACTCAAAGAGCAATCGATGCGGTATCAGCTAAGTATCAAGAAGACCTTGCCGCGCTGGAAGGGAGCACGGATAGGATGATTGCTGATTTGCGTAGCGACAATAAGCGGTGGCGCGTCAGAGGCAAAACTACCgg                    >  1:613096/1‑301 (MQ=255)`  
                                                                                                                                                                                                                                                                                           `gTTAACGGGAGGAATTATGCTAGAATTTTTACGTAAGCTAATCCCTTGGGTTCTCGCTGGGATGCTATTCGGGTTAGGATGGCATCTAGGGTCAGACTCAATGGACGCTAAATGGAAACAGGAGGTACACAATGAGTACGTTAAGAGAGTTTAGGCTGCGAAGAGCACTCAAAGAGCAATCGATGCGGTATCTGCTAAGTATCAAGAAGACCTTGCCGCGCTGGAAGGGAGCACTGATAGGATTATTTCTGATTTGCGTAGCGACAATAAGCGGTTGCGCGTCAGAGTCAAAACTACCGGa                   <  1:757633/301‑1 (MQ=255)`  
                                                                                                                                                                                                                                                                                             `tAACGGGAGGCATTACGCTAGAATTTTTACGTAAGCTAATCCCTTTGGTTCTCGCTGGGATGCTATTCGGGTTAGGATGGCATCTAGGGTCAGACTCAATTGACGCTAAATGGAAACAGGAGGTACACAATGAGTACGTTAAGAGAGTTGAGGCTGCGAAGAGCACTCAAAGAGCAATCGATGCGGTATCTGCTAAGTATCAAGAAGACCTTGCCGCGCTGGAAGGGAGCACTGATAGGATTATTTCTGATTTGCGTAGCGACAATAAGCGGTTGCGCGTCAGAGTCAAAACTACCGGAAc                 <  1:283557/301‑1 (MQ=255)`  
                                                                                                                                                                                                                                                                                                      `gCATTATGCTAGAATTTTTACGTAAGCTAATCCCTTGGGTTCTCGCTGGGATGCTATTCGGGTTAGGATGGCATCTAGGGTCAGACTCAATGGACGCTAAATGGAAACAGGAGGTACACAATGAGTACGTTAAGAGAGTTGAGGCTGCGAAGAGCACTCAAAGAGCAATCGATGCGGTATCTGCTAAGTATCAAGAAGACCTTGCCGCGCTGGAAGGGAGCACTGATAGGATTATTTCTGATTTGCGTAGCGACAATAAGCGGTTGCGCGTCAGAGTCAAAACTACTGGAACCTCCGATgg        >  1:547614/1‑301 (MQ=255)`  
                                                                                                                                                                                                                                                                                                        `agtATGCTAGAATTTTTACGTAAGCTAATCCCTTTGTTTCTCGCTGGGATGCTAGTCGGGTTAGGATGCCATCTAGGGTCAGACTCAATGGACGCTAAATGGAAACAGGCGGTACAGAATGAGTACGTTAAGAGAGTTGAGGCTGCTAAGAGCACTCAAAGAGCAATCGATGCGGTATCTGCTAAGTATCAAGACGACCTTGCCGCGCTGGAAGGGAGCACTGCTAGGATTATTTCTGATTTGCGTAGCGACAATAAGCGGTTGCGCGTCAGAGTCAAAACTACAGGAACCTCCGATGGTc      <  1:818005/299‑1 (MQ=255)`  

                                                                                                                                                                                                                                                                                                            `|`                                                                                                                                                                                                                                                                                                              
`GCTTGCGGACCTTTCGGACCATGAGCGTCGCTCTCCGCAACTCTATAATGCTATTAACAAACTGTTAGACCGCCACAAGTTCCAGATTGGTAAGTTGCAGCCGGATGTTCACATCTTAGGTGGCCTTGCTGGTGCTCTTGAAGAGTACAAAGAGAAAGTCGGTGATAACGGTCTTACGGATGATGATATTTACACATTACAGTGATATACTCAAGGCCACTACAGATAGTGGTCTTTATGGATGTCATTGTCTATACGAGATGCTCCTACGTGAAATCTGAAAGTTAACGGGAGGCATTATGCTAGAATTTTTACGTAAGCTAATCCCTTGGGTTCTCGCTGGGATGCTATTCGGGTTAGGATGGCATCTAGGGTCAGACTCAATGGACGCTAAATGGAAACAGGAGGTACACAATGAGTACGTTAAGAGAGTTGAGGCTGCGAAGAGCACTCAAAGAGCAATCGATGCGGTATCTGCTAAGTATCAAGAAGACCTTGCCGCGCTGGAAGGGAGCACTGATAGGATTATTTCTGATTTGCGTAGCGACAATAAGCGGTTGCGCGTCAGAGTCAAAACTACCGGAACCTCCGATGGTCAGTG  >  NC_001604.1/36618‑37218`

  

Alignment Legend

Aligned base mismatch/match (shaded by quality score): `ATCG/ATCG` `< 3 ≤` `ATCG/ATCG` `< 11 ≤` `ATCG/ATCG` `< 25 ≤` `ATCG/ATCG` `< 36 ≤` `ATCG/ATCG` `< 38 ≤` `ATCG/ATCG`

Unaligned base: `atcg`    Masked matching base: `atcg`    Alignment gap: `‑`    Deleted base: `‑`
